# Thermoresponsive Poly(*N*,*N*-diethylacrylamide-*co*-glycidyl methacrylate) Copolymers and Its Catalytically Active α-Chymotrypsin Bioconjugate with Enhanced Enzyme Stability

**DOI:** 10.3390/polym13060987

**Published:** 2021-03-23

**Authors:** György Kasza, Tímea Stumphauser, Márk Bisztrán, Györgyi Szarka, Imre Hegedüs, Endre Nagy, Béla Iván

**Affiliations:** 1 Polymer Chemistry Research Group, Institute of Materials and Environment Chemistry, Research Centre for Natural Sciences, Magyar tudósok körútja 2., H-1117 Budapest, Hungary; stumphauser.timea@ttk.hu (T.S.); bisztmark@gmail.com (M.B.); szarka.gyorgyi@ttk.hu (G.S.); 2 Chemical and Biochemical Procedures Laboratory, Institute of Biomolecular and Chemical Engineering, Faculty of Engineering, University of Pannonia, Egyetem u. 10, H-8200 Veszprém, Hungary; hegedus@mukki.richem.hu (I.H.); nagy@mukki.richem.hu (E.N.); 3 Department of Biophysics and Radiation Biology, Semmelweis University, Tűzoltó u. 37–47, H-1094 Budapest, Hungary

**Keywords:** poly(*N*,*N*-diethylacrylamide), glycidyl methacrylate, thermoresponsive copolymer, α-chymotrypsin, polymer-enzyme conjugate nanoparticle

## Abstract

Responsive (smart, intelligent, adaptive) polymers have been widely explored for a variety of advanced applications in recent years. The thermoresponsive poly(*N*,*N*-diethylacrylamide) (PDEAAm), which has a better biocompatibility than the widely investigated poly(*N*,*N*-isopropylacrylamide), has gained increased interest in recent years. In this paper, the successful synthesis, characterization, and bioconjugation of a novel thermoresponsive copolymer, poly(*N*,*N*-diethylacrylamide-*co*-glycidyl methacrylate) (P(DEAAm-*co*-GMA)), obtained by free radical copolymerization with various comonomer contents and monomer/initiator ratios are reported. It was found that all the investigated copolymers possess LCST-type thermoresponsive behavior with small extent of hysteresis, and the critical solution temperatures (CST), i.e., the cloud and clearing points, decrease linearly with increasing GMA content of these copolymers. The P(DEAAm-*co*-GMA) copolymer with pendant epoxy groups was found to conjugate efficiently with α-chymotrypsin in a direct, one-step reaction, leading to enzyme–polymer nanoparticle (EPNP) with average size of 56.9 nm. This EPNP also shows reversible thermoresponsive behavior with somewhat higher critical solution temperature than that of the unreacted P(DEAAm-*co*-GMA). Although the catalytic activity of the enzyme–polymer nanoconjugate is lower than that of the native enzyme, the results of the enzyme activity investigations prove that the pH and thermal stability of the enzyme is significantly enhanced by conjugation the with P(DEAAm-*co*-GMA) copolymer.

## 1. Introduction

Today, polymers with special, advanced properties and targeted functionalities, such as responsive (smart, intelligent, adaptive) polymers and macromolecules with well-defined array of functional groups belong to the most intensively investigated fields of polymer science and technology. Reactive functionalities in polymer chains can be introduced either along the chains (pendant functionalities) and/or at the chain ends (terminal functionalities). These functional polymers can be applied in various fields of polymer material science, technology, and industry, such as crosslinkers, chain extenders, and as building blocks of complex macromolecular assemblies [1,2,3,4], and life sciences and biotechnology as well, such as targeting delivery [5,6], biological sensors [7], receptors [8], and surfaces to control cell behavior [9]. Polymers with epoxide or glycidyl functional groups are among the most versatile functional polymeric materials for such purposes, because they can react with numerous nucleophiles, such as amines, thiols, phenols, carboxylic acids, or anhydrides via ring-opening reactions [10,11,12,13,14]. Epoxide functional polymers can be obtained by postmodification of double bond containing side or end group(s) of polymers [15,16,17,18,19,20,21], but such macromolecules can also be synthesized by copolymerizations with epoxy group containing monomers. Undoubtedly, glycidyl methacrylate (GMA) is the most investigated and used monomer to obtain functional macromolecules with epoxy side groups, but other epoxide-containing monomers were also studied, such as 4-vinylphenyl glycidyl ether [22]. Previously, the copolymerization of GMA with numerous monomers, e.g., 3-methylthienyl methacrylate [23], trimethylolpropane trimethacrylate [24], sulfobetaine methacrylate [25], ethylene–methyl acrylate [26], styrene [27,28], 2-hydroxyethyl methacrylate [29], by various polymerization techniques, such as free radical polymerization, ATRP, NMP, RAFT, etc., was widely investigated. These functional materials were successfully applied for several purposes, for instance, medical devices [25,29], compatibilizing agent [26], and metal ion absorbers [30,31,32].

Among responsive materials, LCST-type (LCST = lower critical solution temperature) and UCST-type (UCST = upper critical solution temperature) thermally responsive polymers belong to a unique class of smart materials with broad application possibilities ranging from nanotechnologies, oil recovery [33,34,35], to biomaterials, tissue engineering scaffolds [36,37], intelligent drug release assemblies [36,38,39], sensors [40,41], self-healing structures [42,43], responsive hybrid materials [44,45], etc. As to the use of the LCST and UCST terminology, it has to be noted that most of the authors still report incorrectly the result of a single-point measurement as LCST or UCST, i.e., the result of only one given condition with one single polymer concentration, one single heating/cooling rate and wavelength for cloud point and clearing point determination is claimed misleadingly as LCST or UCST. In contrast, the LCST or UCST are defined as the minimum or maximum, respectively, of the polymer concentration versus critical solution temperature (CST) curves, and not the single CST of a certain selected condition in terms of polymer concentration, heating/cooling rates, and wavelength for cloud point and cooling point determination. Hence, for LCST and UCST determination, the full CST versus polymer concentration (mass fraction or volume fraction) relationship should be measured, and the resulting minimum or maximum of such curves should be reported as LCST or UCST, respectively. Therefore, recently a standardization of the conditions for the measurements of CST in order to obtain comparable results of the laboratories worldwide was proposed on the basis of systematic investigations on the effect of the experimental conditions on the CST of poly(*N*-isopropylacrylamide) (PNIPAAm) solutions [46,47].

Undisputedly, poly(*N*-isopropylacrylamide) has been the most investigated temperature-responsive polymer since the first report of its LCST-type behavior (see, e.g., Refs. [46,47,48,49,50,51,52,53,54,55,56,57,58,59,60,61,62,63] and references therein). Recently, intensive research has been focused on how to control the critical solution temperature (CST) of thermoresponsive polymers by using other monomers than NIPAAm (e.g., other acrylamides and *N*-vinyl lactams), by copolymerization with common monomers, especially with functional monomers, which can further increase the range of potential applications. Although NIPAAm-GMA copolymers were already synthesized and investigated for a variety of purposes [56,57,58,59,60,61,62,63], much less attention was paid to other GMA containing thermoresponsive polymers in the past. Recently, various *N*-vinyl lactam monomers were copolymerized with GMA, and the resulting copolymers were successfully applied as robust building blocks for protein conjugation, and the biohybrid nanogels of these copolymers exhibited significantly enhanced resistance against harsh storing conditions, chaotropic agents, and organic solvents [64].

It is interesting to note that due to the better biocompatibility of the LCST-type thermoresponsive poly(*N*,*N*-diethylacrylamide) (PDEAAm) than that of PNIPAAm [65], investigations in relation to the responsive and biocompatible behavior of PDEAAm, its derivatives, and gels have gained increased attention only in recent years (see, e.g., Refs. [66,67,68,69,70,71,72,73,74,75,76,77,78,79,80,81,82,83,84,85,86,87,88,89,90,91,92] and references therein). It should also be mentioned that aqueous PDEAAm solutions possess similar critical solution temperatures (CST) [72,93,94,95,96] in the range of ~25–40 °C than that of PNIPAAm [46,55]. On the other hand, although some block copolymers with PDEAAm and poly(glycidyl methacrylate) (PGMA) segments have been prepared and studied [91,92], random copolymers of DEAAm with GMA, which provides reactive pendant epoxy functionalities for the thermoresponsive PDEAAm, and its utilization for polymer-based protein engineering have not been reported so far according to the best of our knowledge.

Polymer-based protein engineering mainly focuses on the synthesis, characterization, and applications of conjugates of proteins, especially enzymes, with polymers for various purposes, such as stability improvement, better biodistribution, biocatalytic syntheses, purification, recovery, etc. (see, e.g., Refs. [97,98,99,100,101,102,103,104,105,106,107,108,109,110,111,112,113] and references therein). Among enzymes, α-chymotrypsin (CT), a peptide bond cleaving serine protease enzyme, is one of the most widely investigated proteins in terms of its bioconjugation with a variety of polymers and applications in bioengineering [106,114,115,116,117,118,119,120]. In general, attachment of polymer chains by covalent bonds to CT and other proteins as well can be carried out by either grafting from and grafting onto, and rarely by grafting through as well. Grafting from involves the functionalization of the protein with functional groups suitable for initiating the desired polymerization of selected monomers, usually by a living polymerization process [97,98,99,100,101,102,103,104,105,106,107]. This two-step or multi-step laborious and time-consuming process requires various reagents, in many cases toxic compounds (e.g., copper salts and complex forming amines for quasiliving atom transfer radical polymerization), relatively high temperatures that may deactivate the enzymes, and vigorous purification steps [99,100,101,102,103,104,105,106,107]. Grafting onto takes place by reacting the protein with pre-synthesized functional, mainly endfunctional, polymers, including the widely applied PEGylation with terminally functional PEGs, usually in two or more steps (see, e.g., [97,98,99,100,101,102,113] and references therein). Conjugation of proteins, especially enzymes, with thermoresponsive polymers offers additional unique possibilities for switching enzyme activity, efficient purification, enzyme recovery, etc., based on the precipitation of such polymer assemblies above their critical solution temperature [97,106,107,108,112]. As to grafting onto proteins with epoxy group containing polymers, only few examples can be found in the literature [111,114,115]. Recently, a grafting through approach of an enzyme macromonomer, functionalized with glycidyl methacrylate, was also reported [121]. For α-chymotrypsin, the widely applied method for polymer conjugation with epoxy-functional polymers involves amination of the epoxy group with a diamine followed by coupling the resulting amine-functionalized polymer to the enzyme by glutaraldehyde [114,115]. Although poly(*N*-isopropylacrylamide) bioconjugates have been investigated in numerous cases, its relatively large extent of hysteresis due to hydrogen bonding between PNIPAAm chains [46,47] and even full activity loss of the conjugated enzyme [122] may limit its application possibilities. In contrast, the lack of hydrogen bonding between PDEAAm chains may provide unique advantages for bioconjugations with functional PDEAAm. Considering the high reactivity of primary amines with epoxy, especially glycidyl groups, the question arises whether the biocompatible poly(*N*,*N*-diethylacrylamide) with epoxy functionalities can be conjugated to CT, containing 15 primary amine sites, directly in a simple one-step process, and if this were possible, what are the characteristics of such bioconjugates in terms of their size, thermoresponsive behavior, enzymatic activity, and stability. 

Based on the above aspects and unique potentials of the pendant epoxy containing poly(*N*,*N*-diethylacrylamide-*co*-glycidyl methacrylate) (P(DEAAm-*co*-GMA)) copolymers, we aimed at exploring its thermoresponsive property, the one-step conjugation possibility with α-chymotrypsin, and the catalytic activity and stability of such bioconjugates. Herein, we present the results of our investigations on the synthesis of P(DEAAm-*co*-GMA) and on the thermoresponsive behavior, i.e., on the effect of composition of the resulting copolymers on the critical solution temperature, of the resulting copolymers. In addition, we also report on the utilization of the epoxy functionalities of P(DEAAm-*co*-GMA) for the one-step preparation of enzyme–polymer nanoparticles (EPNP) by conjugation with α-chymotrypsin (CT), its thermoresponsive characteristics, thermal and pH stability, and the enzymatic catalytic behavior of these new bioconjugates.

## 2. Materials and Methods

### 2.1. Materials

Glycidyl methacrylate and *N*,*N*-diethylacrylamide (both from Sigma-Aldrich, St. Louis, MO, USA) were freshly distilled under reduced pressure prior to use. 2,2′-Azoisobutyronitrile (AIBN, 98%, Sigma-Aldrich, St. Louis, MO, USA) was recrystallized from hexane and methanol twice, respectively. Tetrahydrofuran (THF, >99%, Molar Chemicals, Halásztelek, Hungary) was refluxed over LiAlH_4_, distilled, and was kept under nitrogen until its use. Diethyl ether and methanol (>99%, Molar Chemicals, Halásztelek, Hungary), PBS (pH = 7.4) and phosphate buffers (pH = 6; 7; 7.8; 8; 9 both from Sigma-Aldrich, St. Louis, MO, USA) were used without further purification. α-Chymotrypsin and *N*-benzoyl-L-tyrosine ethyl ester (BTEE, 99%) were purchased from Sigma-Aldrich and were used as received.

### 2.2. Synthesis Methods

#### 2.2.1. Synthesis of PDEAAm Homopolymer and P(DEAAm-*co*-GMA) Copolymers by Free Radical Polymerization

Poly(*N*,*N*-diethylacrylamide-*co*-glycidyl methacrylate) (P(DEAAm-*co*-GMA)) copolymers were prepared by free radical copolymerization initiated by AIBN with various initiator/monomer molar ratios (1:100 and 1:200) and comonomer contents (5 and 10 mol% GMA). A typical copolymer synthesis is described below. In the case, when the molar ratio of the components, i.e., AIBN:DEAAm:GMA was 1:95:5, first 1.02 mL of DEAAm (7.43 mmol) and 0.052 mL of GMA (0.39 mmol) were charged into sealed round bottom flask, and the monomers were dissolved in 9.5 mL of THF. This solution was deoxygenized by bubbling with argon for 20 min. Then the reaction mixture was warmed to 60 °C, and 0.5 mL AIBN stock solution (25.75 mg/mL, 0.078 mmol) was added. After stirring for 18 h, the resulting polymer was precipitated twice from THF solution in hexane and filtered. Finally, the product was dried in vacuum at 60 °C until constant weight. The poly(*N*,*N*-diethylacrylamide) (PDEAAm) homopolymer was synthesized by the same method using 100:1 monomer/initiator ratio. 

#### 2.2.2. Synthesis of Enzyme–Copolymer Nanoparticle (EPNP)

One selected P(DEAAm-*co*-GMA) copolymer (Sample C) was measured (15.85 mg) into a glass vial and dissolved in 2 mL water. Then, 3 mL of α-chymotrypsin stock solution (10.14 mg/mL in water) was added dropwise to the stirred polymer solution by a syringe pump (dosing rate was 1.5 mL/h). The reaction mixture was stirred overnight at room temperature. Subsequently, the reaction mixture was dialyzed (MWCO = 25 kDa) for three days against water, which was refreshed twice daily. Then, the purified dry product was obtained by lyophilization.

### 2.3. Characterization

#### 2.3.1. Gel Permeation Chromatography (GPC)

Average molecular weights and molecular weight distributions (dispersity index, *Đ*) of the produced polymers were determined by GPC. The GPC was equipped with differential refractive index detector (Agilent 390, Agilent Technologies, Santa Clara, CA, USA), three 5 μm particle size Waters Styragel (columns (HR1, HR2 and HR4) and with a Waters Styragel guard column (both form Waters, Milford, MA, USA) thermostated at 35 °C. THF was used as eluent with a flow rate of 0.3 mL/min. The average molecular weights and *Đ* were determined by using conventional calibration based on linear polystyrene standards (from PSS Polymer Standards Services GmbH, Mainz, Germany).

#### 2.3.2. ^1^H NMR Spectroscopy

The ratio of the incorporated comonomers was determined by ^1^H NMR measurements. The analysis was performed on Bruker Advance 500 (Bruker, Billerica, MA, USA) equipment operating at 500 MHz ^1^H frequency in CDCl_3_ at 30 °C.

#### 2.3.3. Thermoresponsive Behavior

The transmittance versus temperature curves for obtaining the critical solution temperatures (*T*_C_), i.e., the cloud point (*T*_CP_) and the clearing point (*T*_CL_) were measured by a UV–Vis spectrophotometer (Jasco V-650, JASCO Corporation, Tokyo, Japan) equipped with Jasco MCB-100 (JASCO Corporation, Tokyo, Japan) mini circulation bath and Peltier thermostat. Standard 1 cm × 1 cm cuvettes were used for these measurements. Deionised water was used as reference and solvent. The polymer and the enzyme–polymer nanoparticle solutions (1 mg/mL) were heated and then cooled in the temperature range of 15 to 50 °C with 0.2 °C/min heating/cooling rate and the transmittance was recorded at 488 nm according to recent studies on the standardization of measurements for the determination of the critical solution temperatures [46,47]. The inflection points of the transmittance–temperature curves were taken as both the *T*_CP_ and *T*_CL_ values.

#### 2.3.4. Dynamic Light Scattering (DLS)

The average hydrodynamic diameter and dispersity of the obtained enzyme–polymer nanoparticle and the applied copolymer as well as the α-chymotrypsin were determined by a dynamic light scattering (DLS) system (Malvern Zetasizer Nano ZS, Malvern, UK). The measurements were carried out at 25 °C, and the concentrations of the samples were 1 mg/mL in PBS (pH = 7.4).

#### 2.3.5. Quantification of the Enzyme Content in the Nanoparticles

The enzyme content of the nanoparticles was determined by UV-Vis spectroscopy measurements. The absorbance of the nanoparticle aqueous solution (1 mg/mL) was recorded by UV-Vis spectrophotometer (Jasco V-650, JASCO Corporation, Tokyo, Japan) equipped with Jasco MCB-100 mini circulation bath and Peltier thermostat at 25 °C in the 200–355 nm range. The enzyme content of the nanoparticles was evaluated on basis of an α-chymotrypsin calibration curve at 283 nm, where the polymer has no absorbance.

#### 2.3.6. Catalytic Activity Assay

The catalytic activity of α-chymotrypsin and the produced enzyme–polymer nanoparticle (EPNP) was investigated by UV-Vis spectroscopy assay [123]. In this assay, the transformation of the substrate *N*-benzoyl-l-tyrosine ethyl ester (BTEE) to *N*-benzoyl-*l*-tyrosine via enzymatic hydrolysis was followed by spectroscopy measurements at 256 nm. The measurements were carried out in 3 mL quartz cuvettes, where 1.5 mL buffer (pH = 6; 7; 7.4 (PBS); 7.8; 8; 9), 1.4 mL BTEE stock solution (prepared by dissolving 74.3 mg BTEE in 126.8 mL methanol and adjusted by water to 200 mL in a volumetric flask) and 0.1 mL enzyme or enzyme–polymer nanoparticle solution were mixed (the enzyme concentration of the enzyme stock solution was 0.1 mg/mL). The increment in the absorbance at 256 nm was measured for five minutes with 10 s delays. Three independent measurements were carried out with every sample with varying pH at 25 °C, and good reproducibility was observed in each case. To eliminate the error due to the autohydrolysis of BTEE, measurements were performed by using a blank at every pH with the replacement of the enzyme solutions with distilled water. The reference was distilled water. For investigations of the thermal stability of the enzyme–polymer nanoparticles, the α-chymotrypsin and the nanoparticle solutions with enzyme concentrations of 0.1 mg/mL were thermostated at 45 °C. After predetermined time (5, 15, 30, 60, 120 min) of such thermal treatment, 0.1 mL samples were withdrawn and allowed to cool to room temperature for 5 min. Then the catalytic activity was measured by the method described above using PBS buffer (pH = 7.4) at 25 °C. The enzymatic activity was calculated with the following equation:

Activity = ((Δ*A*_T_ − Δ*A*_B_) *V*_T_*d*_f_)/(0.964 vs. *c*_e_), (1)

where Δ*A*_T_ and Δ*A*_B_ are the maximum rate of increase in the absorbance in one minute for the test sample and blank, respectively, *V*_T_ is the total volume (3 mL), d_f_ is the dilution factor (30), 0.964 is the millimolar extinction coefficient of BTEE at 256 nm, vs. is the sample volume (0.1 mL), and *c*_e_ is the enzyme concentration (0.1 mg/mL).

## 3. Results and Discussion

**Scheme 1:**
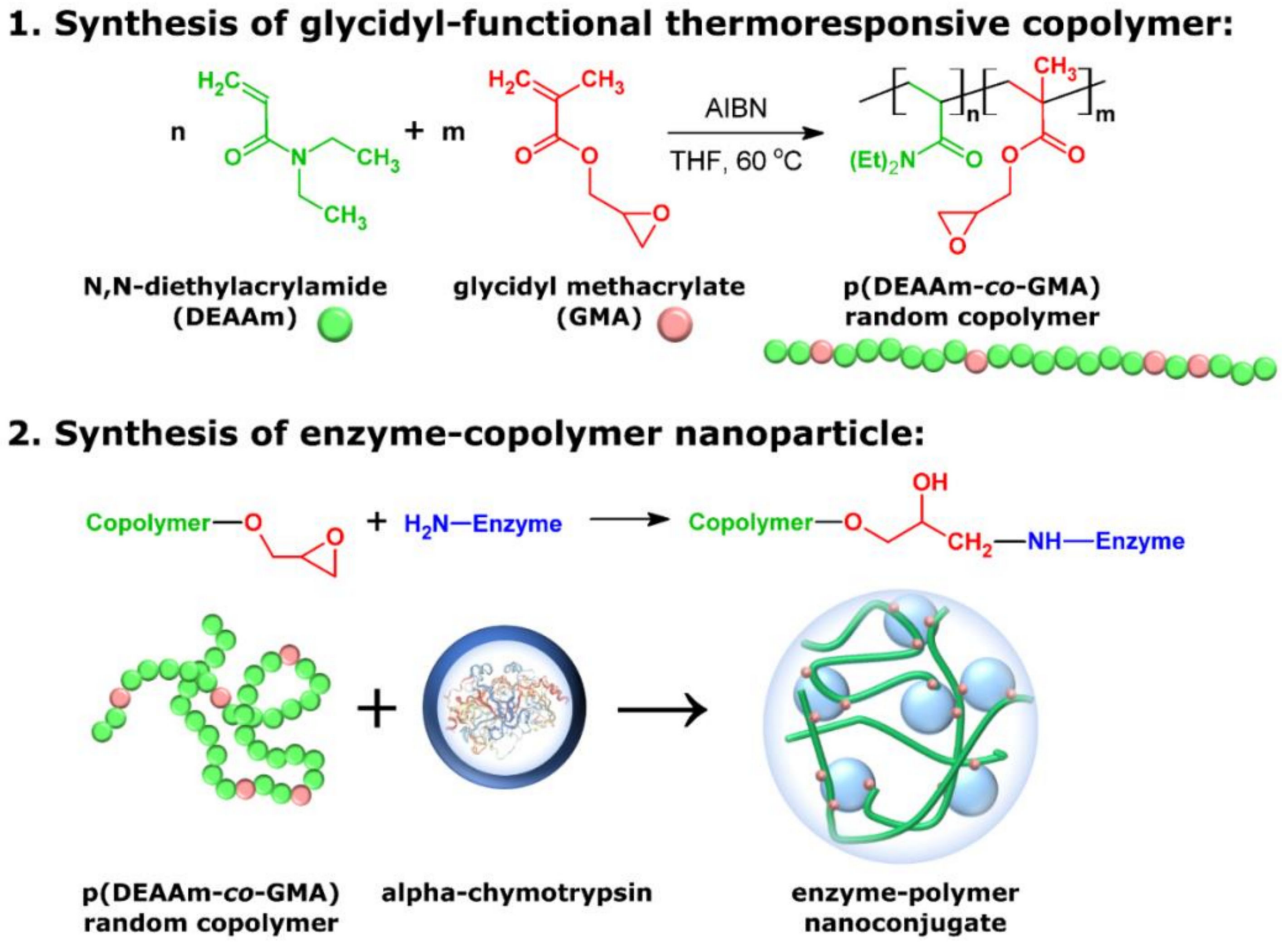
Synthesis of glycidyl-functional poly(*N*,*N*-diethylacrylamide-*co*-glycidyl methacrylate) copolymers via free radical copolymerization (**1**) and the design of enzyme–polymer nanoconjugate with α-chymotrypsin (**2**).

**Figure 1:**
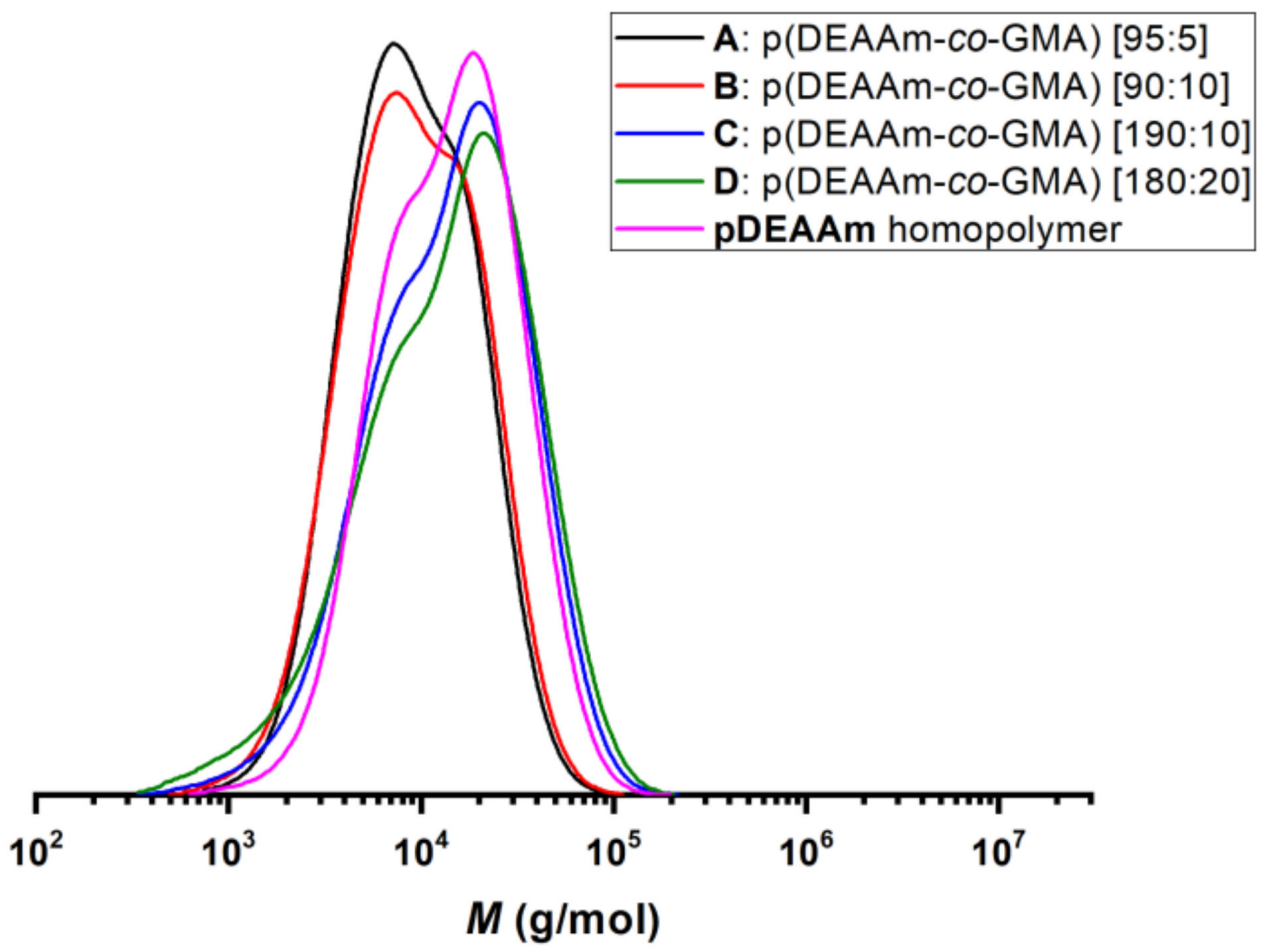
Molar mass distribution curves of the P(DEAAm-*co*-GMA) copolymers and PDEAAm homopolymer.

As displayed in Scheme 1, we aimed at synthesizing poly(*N*,*N*-diethylacrylamide-*co*-glycidyl methacrylate) (P(DEAAm-*co*-GMA)), preparing α-chymotrypsin-P(DEAAm-*co*-GMA) bioconjugate by the utilization of the reactive pendant epoxy functional groups of this copolymer, and characterization of the resulting copolymers and enzyme–polymer nanoparticles (EPNP) in terms of their thermoresponsive behavior, enzyme activity, and stability. The P(DEAAm-*co*-GMA) copolymers were synthesized by free radical copolymerization of PDEAAm and GMA with AIBN as radical initiator by using two different monomer/initiator ratios (100 and 200) with two different comonomer contents (5 and 10 mol%). PDEAAm homopolymer was also prepared with 100:1 monomer/initiator ratio under identical conditions to that of the copolymer syntheses. As shown in Table 1, polymers with relatively high yields in the range of ~60–87% were obtained. The molecular mass distributions (MMD) of the resulting polymers, displayed in Figure 1, were determined by GPC analysis (the GPC chromatograms are shown in Figure S1 in the Supplementary Materials). As expected, these MMD curves in Figure 1, and the number average molecular weight (M_n_) and the peak molecular weight values (M_p_) in Table 1 clearly indicate that P(DEAAm-*co*-GMA) copolymers with higher molecular masses are formed with higher monomer/initiator ratios.

**Figure 2:**
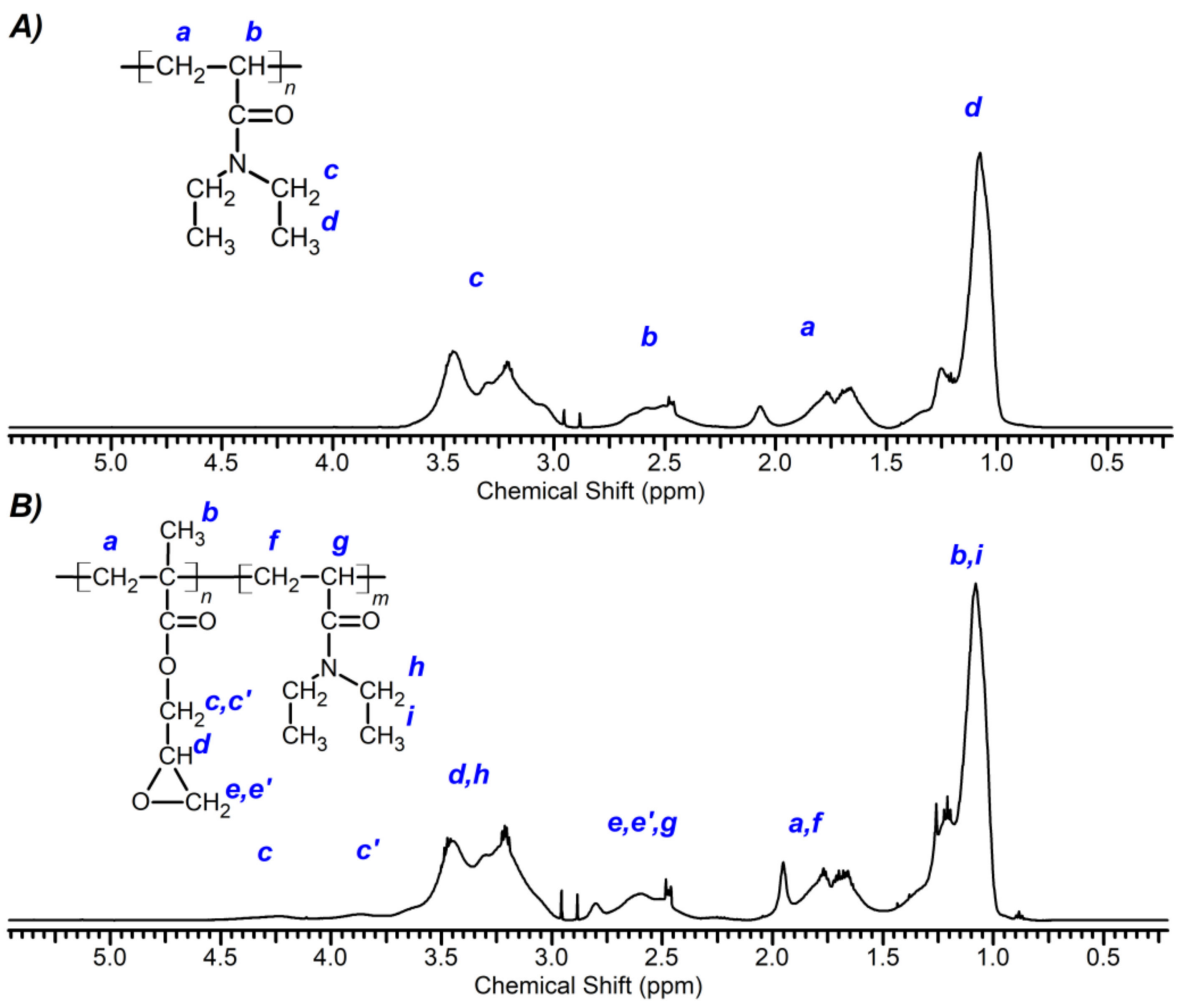
^1^H NMR spectra of the PDEAAm homopolymer (**A**) and the P(DEAAm-*co*-GMA) copolymer (Sample A, molar feed ratio AIBN:DEAAm:GMA = 1:95:5) (**B**).

The compositions of the P(DEAAm-*co*-GMA) copolymers were determined by ^1^H NMR spectroscopy. Comparing the ^1^H NMR spectra of the PDEAAm homopolymer (Figure 2A) with that of the P(DEAAm-*co*-GMA) copolymers (Figure 2B and Figures S2–S4), it can be seen that with the exception of the chemical shifts of the methylene group next to the epoxy group in the GMA monomer units (d*CH*_2_ 3.7–4.1 and 4.1–4.6 ppm), the rest of the signals overlap with that of the PDEAAm homopolymer. This allows the determination of the composition of the P(DEAAm-*co*-GMA) copolymers by the integral values of the ^1^H NMR signals. As shown in Table 2, the DEAAm/GMA ratios are smaller in the copolymers than in the feed. This means that the GMA contents in the P(DEAAm-*co*-GMA) copolymers are higher than that in the feed, which means that the reactivity of GMA is higher than that of DEAAm in this copolymerization reaction. This is in line with the reactivity ratios reported for the copolymerization of another alkyl acrylamide, *N*-isopropylacrylamide (NIPAAm), and GMA, according to which r_1_ = 0.39 and r_2_ = 2.69 [56]. Taking into account the similar structure of NIPAAm and DEAAm, higher reactivity of GMA is expected in the DEAAm-GMA copolymerization process as well, on the one hand. Considering that the product of the r_1_ and r_2_ values of the alkyl acrylamide copolymerization with GMA is in the range of one, random copolymerization occurs in such cases, on the other hand. Thus, it can be concluded that random copolymers of DEAAm and GMA with 5.5–11.4 mol% GMA contents were obtained in the applied copolymerization reactions as shown in Table 2. 

**Figure 3:**
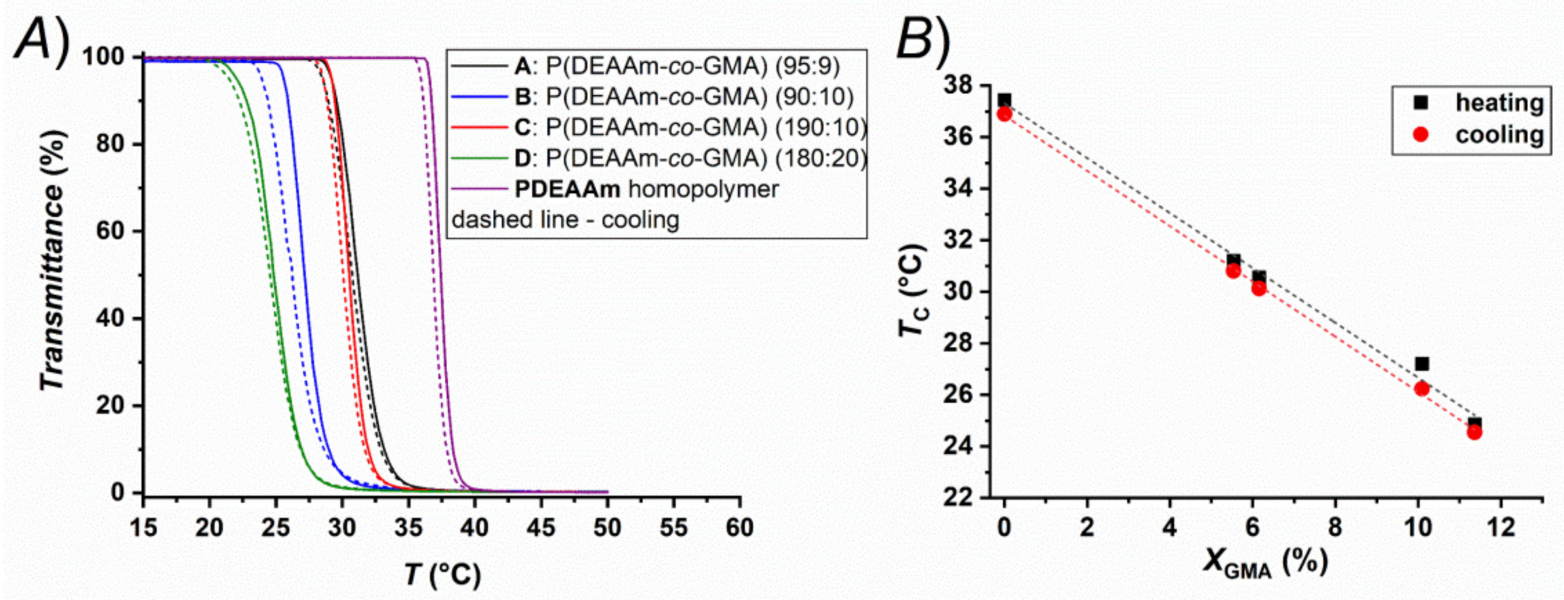
Transmittance vs. temperature curves of the P(DEAAm-*co*-GMA) copolymers and the PDEAAm homopolymer during heating and cooling (**A**) and the cloud points and clearing points as a function of the DEAAm content (**B**).

The thermoresponsive behavior of the P(DEAAm-*co*-GMA) copolymers was investigated by turbidity measurements under the conditions proposed for standardization of the determination of critical solution temperatures of thermoresponsive LCST-type and UCST-type polymers [46,47]. As shown in Figure 3A, the transmittance–temperature curves of the heating and cooling cycles indicate reversible thermoresponsive precipitation–dissolution transitions for both the PDEAAm homopolymer and all the investigated copolymers with relatively small extent of heating–cooling hysteresis due to the lack of hydrogen bond formation between the PDEAAm chains in accordance with previous results [96]. The critical solution temperature (*T*_C_) is defined as the temperature at the inflection point of the transmittance–temperature curves, i.e., the so-called cloud point temperature (*T*_CP_) for heating and the clearing point temperature (*T*_CL_) for cooling. As presented in Figure 3B and Table 2, the critical solution temperatures decrease linearly with decreasing DEAAm, i.e., with increasing GMA content, independent of the molar mass of the copolymers. It should also be noted that incorporating relatively low amounts of GMA in the P(DEAAm-*co*-GMA) copolymers results in significant decrease of the critical solution temperature (*T*_C_) values (from 37.4 °C for the homopolymer to 24.8 °C at 11.4 mol% GMA in the copolymer), and this can be well tuned on the basis of the found linear relationship between *T*_C_ and the composition of the P(DEAAm-*co*-GMA) copolymers. Similar tendency was found for the critical solution temperature versus composition of poly(*N*,*N*-dimethylacrylamide-*co*-glycidyl methacrylate) copolymers but at much higher GMA contents (32–50 mol%) [124].

**Figure 4:**
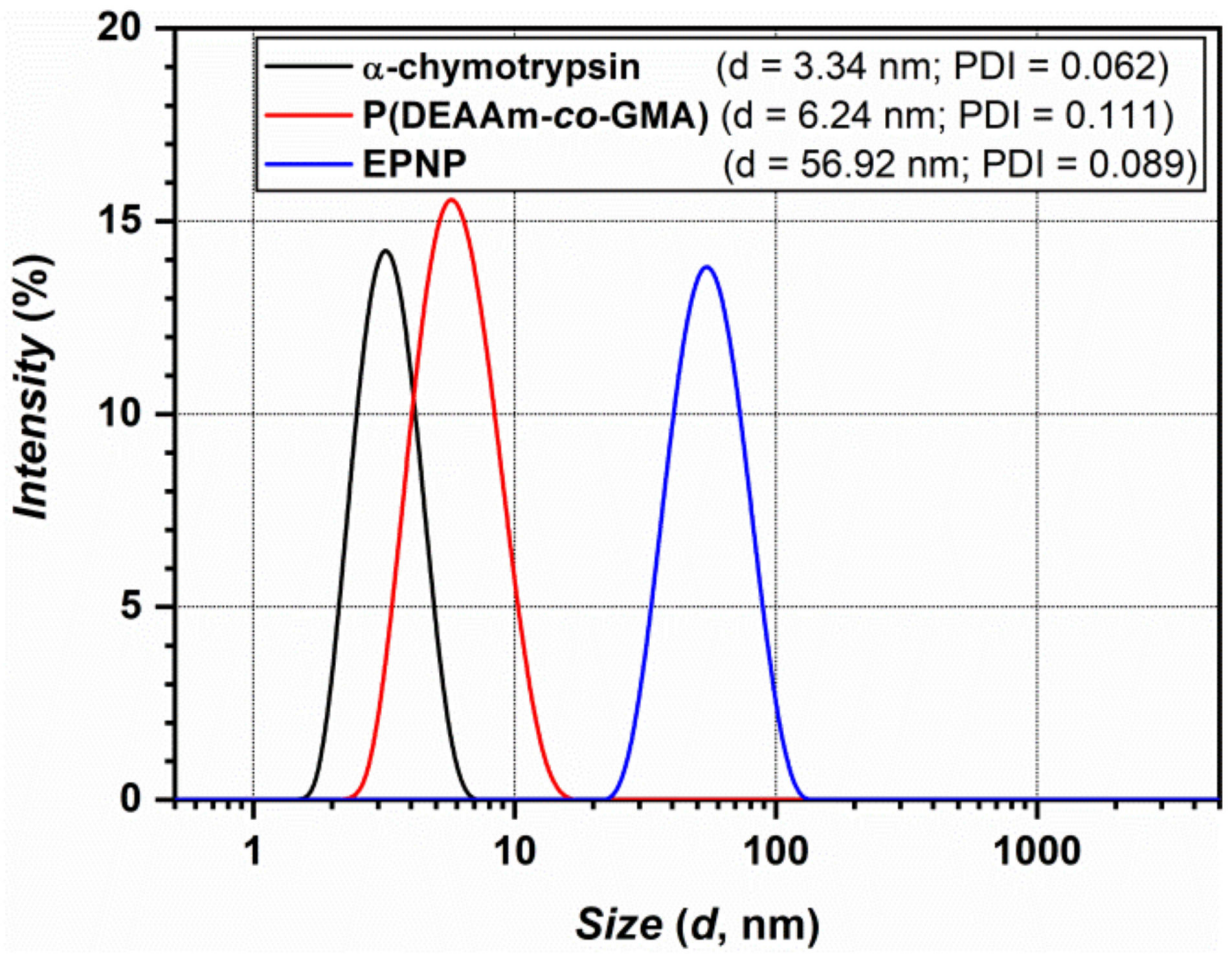
DLS size distribution curves of the α-chymotrypsin (black), the P(DEAAm-*co*-GMA) copolymer (Sample C) (red) and the produced enzyme–polymer conjugate (blue).

One of the intensively investigated application of epoxy(glycidyl)-functionalized polymers is their conjugation with various biomaterials, such as proteins and enzymes. In our work, the applicability of the produced epoxy-functional thermoresponsive P(DEAAm-*co*-GMA) copolymers for bioconjugation was investigated via a direct reaction between α-chymotrypsin, a widely used enzyme, and one selected copolymer (Sample C), as depicted in Scheme 1. Under the conditions described in the Experimental, 22.5 mg dried conjugate was obtained, which means that the yield of the conjugation was 48%. The resulting conjugate was investigated by DLS measurement and the results are compared to that of the starting copolymer sample and the enzyme. The recorded size distribution curves are presented in Figure 4. The size of the α-chymotrypsin is 3.34 nm with low dispersity, which corresponds well to the literature value [125]. The size of the P(DEAAm-*co*-GMA) copolymer is somewhat larger and has broader size distribution (d = 6.24 nm, PDI = 0.11). As observed, the size of the resulting enzyme–copolymer conjugate is in the range of 30–100 nm with average size of 56.9 nm. In addition, peaks do not appear in the range of the size of the reactants, which means that all unreacted copolymer and enzyme was removed by the applied dialysis purification method. These findings provide clear evidence that the designed one-step reaction took place successfully, and enzyme–polymer nanoparticles (EPNPs) are formed in the direct conjugation reaction between the P(DEAAm-*co*-GMA) copolymer and α-chymotrypsin. It has to be noted that usually epoxy containing carriers, i.e., polymers or inorganic particles, are first converted to amine by either treating with ammonia or a diamine, and then the conjugation (coupling) to the enzyme is carried out by glutaraldehyde [114,115]. In contrast to this widely applied two-step conjugation process, the P(DEAAm-*co*-GMA) copolymers enable an efficient one-step conjugation reaction with amine containing proteins and enzymes as proved by our results. This finding may open new routes for a variety of novel protein-polymer conjugations, especially by applying the biocompatible thermoresponsive epoxy-functionalized PDEAAm. 

The enzyme content of the produced enzyme–polymer nanoparticle (EPNP) was determined by UV-Vis spectroscopy. The recorded UV spectra of the EPNP and the unreacted copolymer are presented in Figure S5. As can be seen, the copolymer has no absorbance above 250 nm, but a broad peak appears in the spectrum of the EPNP in the 260–300 nm range due the aromatic side groups of the enzyme component. This also confirms that the enzyme is incorporated into the EPNP. In addition, it allows the determination of the enzyme content as well, because the composition of the EPNP can be determined on the basis of calibration with the enzyme at a selected wavelength, 283 nm in this case (see Figure S6 in the Supplementary Materials). The determined enzyme content is 0.687 mg/mg EPNP, which means that the produced nanoconjugate consists of 68.7% of α-chymotrypsin and 31.3% of P(DEAAm-*co*-GMA) copolymer. Considering the composition of the EPNP gives that the molar ratio of GMA to the enzyme in the conjugate is 5.4, i.e., sufficiently high for coupling of the copolymer even to more than one CT molecule. A rough estimate can also be provided on the average number of the copolymer chains and enzyme molecules in the bioconjugate if it is assumed that the diameter (volume) of the components does not change by conjugation. On the basis of this approximation, the average numbers of the P(DEAAm-*co*-GMA) copolymer and the enzyme in their conjugate are around 6.5 and 5, respectively. 

**Figure 5:**
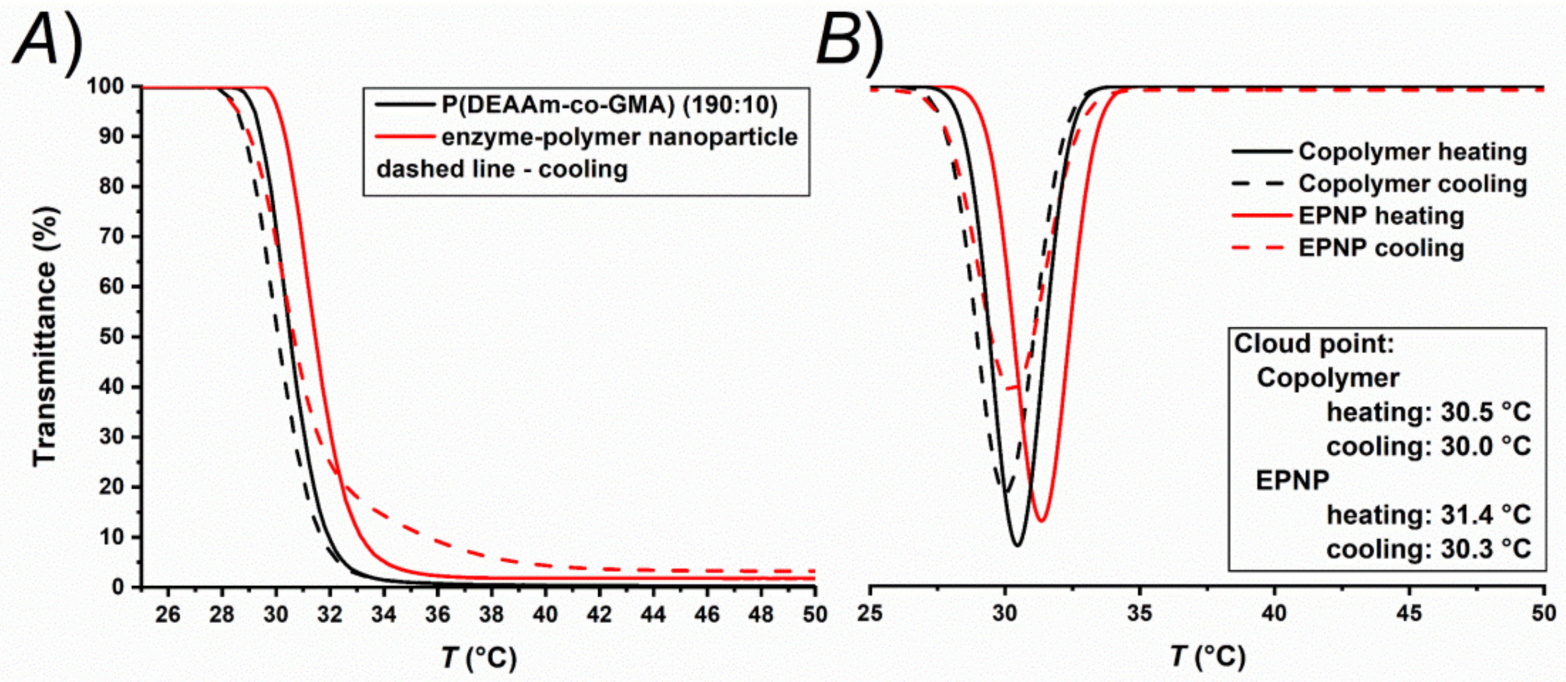
Transmittance vs. temperature curves (**A**) and its first derivatives (**B**) of the copolymer (black) and enzyme–polymer nanoparticle (red) in heating (full line) and cooling cycle (dashed line).

The effect of the conjugation on the thermoresponsive behavior of the EPNP was investigated by turbidimetry. The transmittance versus temperature curves (Figure 5A) and its first derivative (Figure 5B) of the EPNP are plotted and compared to that of the unmodified copolymer. As shown in this Figure, the thermal transition is slightly shifted to higher temperature by the conjugation, but the shape of the curve is similar to that of the unreacted copolymer. In the cooling cycle, the temperature range of the dissolution process is significantly broadened for the EPNP, but it has to be emphasized that the transmittance of the EPNP is returned to its maximum value (100% transmittance) by cooling, indicating that the EPNP preserved the reversible thermoresponsive behavior.

**Figure 6:**
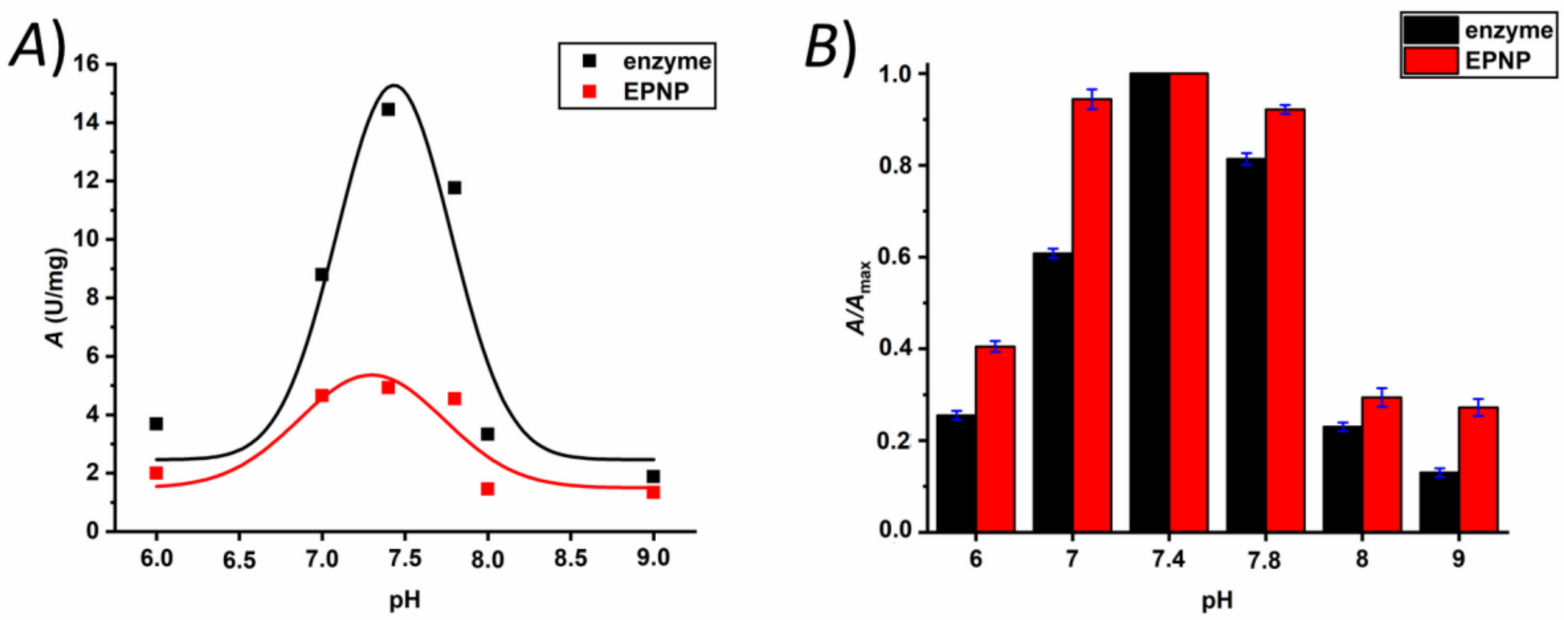
Enzymatic activity (**A**) and the relative activity (**B**) of α-chymotrypsin (black) and the enzyme–polymer nanoparticle (red) as a function of pH.

The applicability of the produced EPNP and the effect of the conjugation on the catalytic activity were investigated by enzymatic activity assay, where the enzymatic hydrolysis of BTEE was followed by UV-Vis spectroscopy at 25 °C in solutions of various pH and after thermal treatment at 45 °C. The enzymatic activity was calculated based on the rate of the conversion of the BTEE substrate. The determined enzymatic activity of the α-chymotrypsin-P(DEAAm-*co*-GMA) EPNP in the 6–9 pH range is presented and compared to the unmodified enzyme in Figure 6. As can be seen in this Figure, the enzymatic activity of the EPNP is significantly lower than that of the native enzyme, but this is a general phenomenon in the case of enzyme conjugates [50,51,52]. This can be explained by decreased accessibility of the substrate to the active pocket of the enzyme in the enzyme–polymer conjugates. The highest enzymatic activity was observed in the pH 7–7.5 range. The pH optimum was determined by the inflection point of the first derivative of the Gauss function fitted on the activity data. As shown in Figure 6A, on the one hand, the pH optimum of the EPNP is slightly lower (pH = 7.3) than that of the native enzyme (pH = 7.4). On the other hand, the activity is greatly decreased even with a slight change in pH in the case of the native α-chymotrypsin, but only a lower extent of change of activity was observed for the EPNP. 

For better understanding, the effect of the pH change on the enzymatic activity, the relative (also called as residual) activity was expressed by the ratio of the measured activity and the maximal activity (Figure 6B). In the case of the native enzyme, the activity is decreased drastically by 0.4 pH change, namely the activity is only 80% and 60% of the maximum at pH 7.8 and 7.0, respectively. In addition, by getting away from the optimum pH value to pH 6 and pH 9, the relative activity is further decreased to ~20%. In contrast, less than 10% activity loss was observed in the pH range of 7–7.8 in the case of the EPNP, and the relative activity was also significantly higher than that of the native enzyme at more extreme pH values. Thus, it can be concluded that the polymer conjugation with P(DEAAm-*co*-GMA) advantageously enhances the pH stability of α-chymotrypsin.

**Figure 7:**
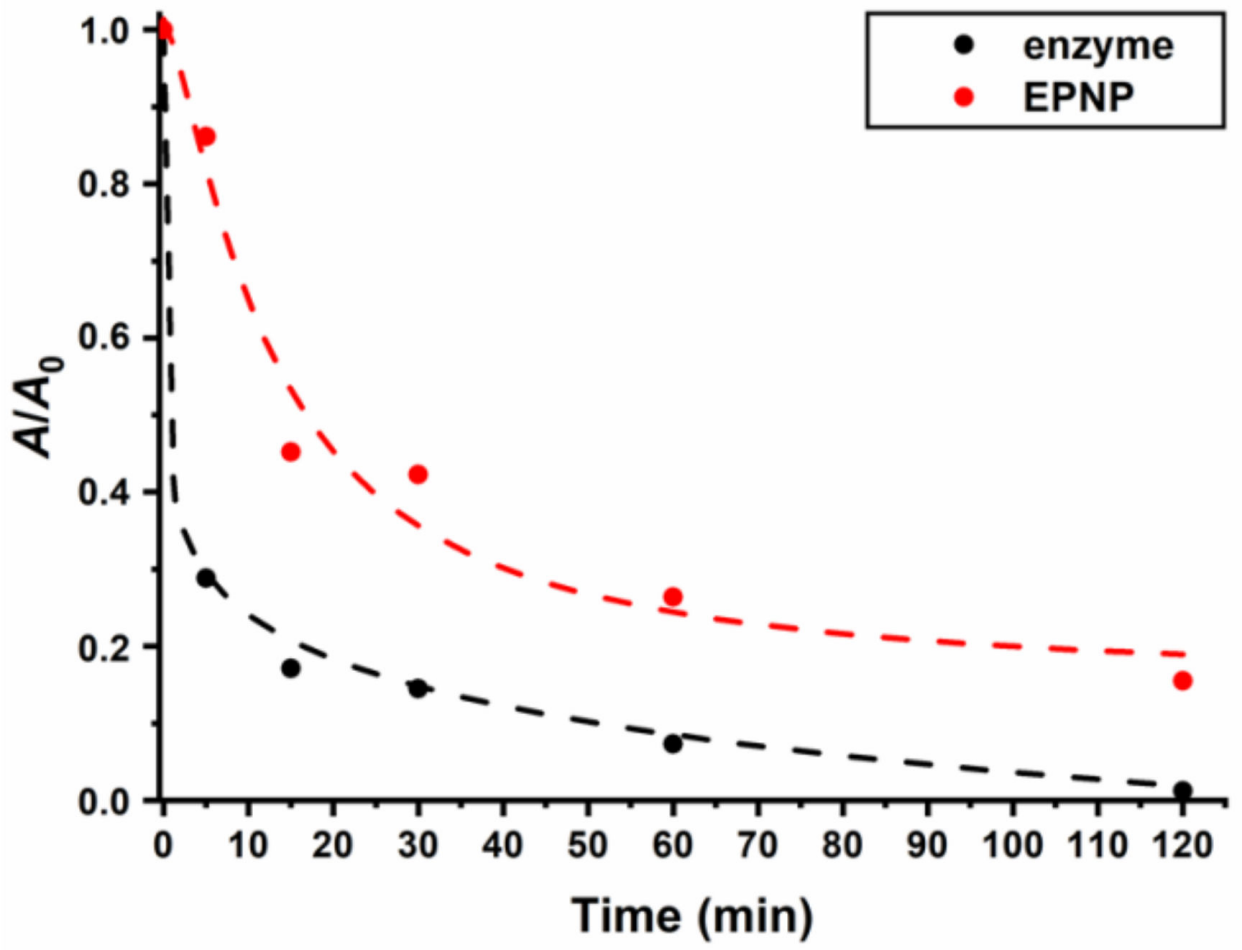
The relative activity of α-chymotrypsin (black) and the CT-P(DEAAm-*co*-GMA) enzyme–polymer nanoparticle (EPNP) (red) at 25 °C as a function of the time of thermal treatment at 45 °C.

The thermal stability of the enzyme–polymer nanoconjugate was also studied. The solution of the EPNP and the native enzyme as well was thermostated at 45 °C, and samples were taken at predetermined treatment times. Because the results of the thermoresponsive investigation of the EPNP shows that the hydrophobic–hydrophilic transition occurs in a wider temperature range during cooling, the withdrawn samples were allowed to cool to room temperature for 5 min before the activity assay measurements at 25 °C. The obtained relative activity plotted as a function of the thermal treatment time is displayed in Figure 7. As can be seen in this Figure, the initial activity of the free α-chymotrypsin decreases to 20% after only 10 min and to 10% after 60 min, and the enzyme becomes completely inactive after 120 min thermal treatment. This finding is in good agreement with results of others [118], according to which native CT loses its activity after thermal treatment at 50 °C for 90 min. It is widely accepted that this caused by the unfolding and inactivation during thermal denaturation of the enzymes. In contrast, the residual activity of the polymer conjugated enzyme in the EPNP is much higher, namely the relative activity is around 85% after five minutes and it is still over 40% after 30 min. Furthermore, the enzymatic activity of EPNP does not fall below 20% even after two hours thermal treatment. These results clearly indicate that the conjugated polymer can reduce the thermal unfolding of the enzyme in the produced nanoconjugate. Hence, it can be concluded that the enzyme–polymer nanoparticle preserves the enzymatic activity after heating, that is, the conjugation with the thermoresponsive P(DEAAm-*co*-GMA) copolymer increases advantageously the thermal stability of α-chymotrypsin.

## 4. Conclusions

Poly(*N*,*N*-diethylacrylamide-*co*-glycidyl methacrylate) (P(DEAAm-*co*-GMA)) copolymers, unreported so far, were successfully synthesized by free radical copolymerization. On the one hand, turbidity measurements revealed that the P(DEAAm-*co*-GMA) copolymers still possess reversible thermoresponsive behavior. It was found that the cloud point and clearing point temperatures of the copolymers are lower than that of the PDEAAm homopolymer and decrease linearly with increasing GMA content in the investigated composition range up to 11 mol% GMA. The reactivity of the epoxy (glycidyl) pendant groups and the applicability of such copolymers were demonstrated by a conjugation reaction with α-chymotrypsin. The formation of nanosized enzyme–polymer conjugates was confirmed by DLS with average diameter of 56.9 nm. In addition, it was also confirmed that not only the P(DEAAm-*co*-GMA) copolymers, but its enzyme–polymer nanoparticles (EPNP) also possess the reversible thermoresponsive behavior. The enzymatic activity of the produced EPNP was investigated at various pH and after 45 °C thermal treatment, and compared to that of the native enzyme. It was found that the activity of the EPNP was lower than that of the free α-chymotrypsin, but the relative activity results proved that the activity of the EPNP is less sensitive to the changes of the pH and the temperature. On the basis of these findings, it can be concluded that the enzyme stability can be significantly enhanced by the polymer conjugation with P(DEAAm-*co*-GMA) copolymers. These findings can be utilized in a variety of applications, e.g., preparation of novel thermoresponsive protein-P(DEAAm-*co*-GMA) bioconjugates with enhanced stability in a one-step process, separation of the products from the thermoresponsive enzyme–polymer conjugates precipitating above its critical solution temperature etc. 

## Figures and Tables

**Table 1:** Yields and GPC results of the PDEAAm homopolymer and P(DEAAm-*co*-GMA) copolymers.

Sample	Molar Feed Ratio AIBN:DEAAm:GMA	Yield %	*M*_n_ (g/mol)	*M*_p_ (g/mol)	*Đ*
A	1:95:5	62.7	6650	7530	1.75
B	1:90:10	69.6	6525	7620	1.88
C	1:190:10	75.6	8620	20,170	2.31
D	1:180:20	86.8	7520	21,470	2.80
PDEAAm	1:100:0	81.5	9820	18,840	1.90

**Table 2:** The DEAAm/GMA molar ratios in the feed and in the P(DEAAm-*co*-GMA) copolymers, the molar percent of the GMA (*X*_GMA_) of the polymers and the cloud point (*T*_CP_) and clearing point (*T*_CL_) measured by turbidimetry.

Sample	DEAAm/GMA Comonomerfeed Ratio	DEAAm/GMA Ratio in the Copolymers *^a^*	*X*_GMA_ (%)	*T*_CP_ (°C)	*T*_CL_ (°C)
A	19:1	17.06:1	5.5	31.2	30.8
B	9:1	8.91:1	10.1	27.2	26.2
C	19:1	15.25:1	6.2	30.6	30.1
D	9:1	7.80:1	11.4	24.8	24.6
PDEAAm	-	-	0	37.4	36.9

*^a^* Determined by ^1^H NMR analysis.

## Data Availability

The data presented in this study are available on request from the corresponding authors.

## Supplementary Materials

The following are available online at https://www.mdpi.com/2073-4360/13/6/987/s1, Figure S1: GPC chromatograms of the P(DEAAm-*co*-GMA) copolymers and PDEAAm homopolymer, Figure S2: ^1^H NMR spectrum of *Sample B* P(DEAAm-*co*-GMA) copolymer (molar feed ratio AIBN:DEAAm:GMA = 1:90:10), Figure S3: ^1^H NMR spectrum of *Sample C* P(DEAAm-*co*-GMA) copolymer (molar feed ratio AIBN:DEAAm:GMA = 1:190:10), Figure S4: ^1^H NMR spectrum of *Sample D* P(DEAAm-*co*-GMA) copolymer (molar feed ratio AIBN:DEAAm:GMA = 1:180:20), Figure S5: UV spectra of the P(DEAAm-*co*-GMA) (Sample *C*, blue) and the produced enzyme–polymer nanoparticle (red), Figure S6: UV spectra of the α-chymotrypsin in the concentration range of 0.033–1 mg/mL (a) and the calibration curve fitted on the absorbance at 283 nm as a function of the enzyme concentration (b), Figure S7: Representative enzymatic activity investigation curves of the absorbance measurement of the enzyme (black) and EPNP (red) in time in different pH solvents (pH = 6 (A); 7 (B); 7.4 (C); 7.8 (D); 8 (E); 9 (F)), Figure S8: Representative curves of the activity measurements of the enzyme (black) and EPNP (red) in PBS buffer after thermostated at 45 °C for 0 min (A), 5 min (B), 15 min (C), 30 min (D), 60 min (E) and 120 min (F).
