# Supplementary material for: Thermoresponsive Poly(N,N-diethylacrylamide-co-glycidyl methacrylate) Copolymers and Its Catalytically Active α-Chymotrypsin Bioconjugate with Enhanced Enzyme Stability
Source: Polymers (Basel). 2021 Mar 23;13(6):987. doi: 10.3390/polym13060987 (PMC8004754; doi:10.3390/polym13060987)
Supplement: Supplementary file 1 [file polymers-13-00987-s001.pdf]

*polymers*

*Supplementary Materials*

# Thermoresponsive Poly(*N*,*N*-diethylacrylamide-*co*-glycidyl methacrylate) Copolymers and Its Catalytically Active $\alpha$-Chymotrypsin Bioconjugate with Enhanced Enzyme Stability

**György Kasza [1,*,†], Tímea Stumphauser [1,†], Márk Bisztrán [1], Györgyi Szarka [1], Imre Hegedüs [2,3], Endre Nagy [2] and Béla Iván [1,*]**

[1] Polymer Chemistry Research Group, Institute of Materials and Environment Chemistry, Research Centre for Natural Sciences, Magyar tudósok körútja 2., H-1117 Budapest, Hungary; stumphauser.timea@ttk.hu (T.S.); bisztmark@gmail.com (M.B.); szarka.gyorgyi@ttk.hu (G.S.)
[2] Chemical and Biochemical Procedures Laboratory, Institute of Biomolecular and Chemical Engineering, Faculty of Engineering, University of Pannonia, Egyetem u. 10, H-8200 Veszprém, Hungary; hegedus@mukki.richem.hu (I.H.); nagy@mukki.richem.hu (E.N.)
[3] Department of Biophysics and Radiation Biology, Semmelweis University, Tűzoltó u. 37–47, H-1094 Budapest, Hungary
* Correspondence: kasza.gyorgy@ttk.hu (G.K.); ivan.bela@ttk.hu (B.I.)
† These authors contributed equally to this work.

**Citation:** Kasza, G.; Stumphauser, T.; Bisztrán M.; Szarka, G.; Hegedüs, I.; Nagy, E.; Ivan, B. Thermoresponsive Poly(*N*,*N*-diethylacrylamide-*co*-glycidyl methacrylate) Copolymers and Its Catalytically Active $\alpha$-Chymotrypsin Bioconjugate with Enhanced Enzyme Stability. *Polymers* **2021**, *13*, 987. https://doi.org/10.3390/polym13060987

Academic Editor: Florian J. Stadler

Received: 1 March 2021
Accepted: 20 March 2021
Published: 23 March 2021

**Publisher's Note:** MDPI stays neutral with regard to jurisdictional claims in published maps and institutional affiliations.

**Copyright:** © 2021 by the authors. Submitted for possible open access publication under the terms and conditions of the Creative Commons Attribution (CC BY) license (http://creativecommons.org/licenses/by/4.0/).

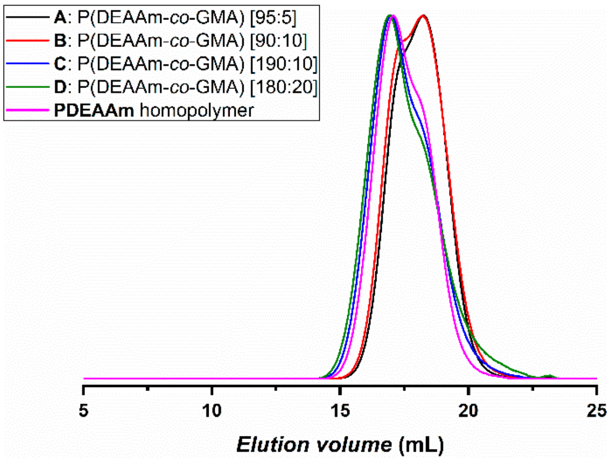


**Figure S1.** GPC chromatograms of the P(DEAAm-*co*-GMA) copolymers and PDEAAm homopolymer.

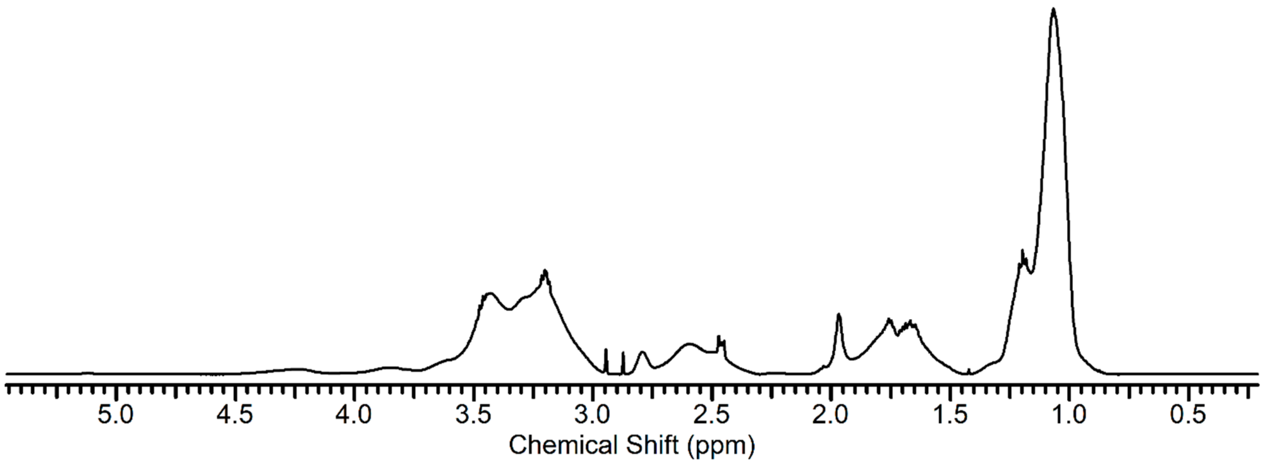


**Figure S2.** $^{1}$H NMR spectrum of *Sample **B*** P(DEAAm-*co*-GMA) copolymer (molar feed ratio AIBN:DEAAm:GMA = 1:90:10).

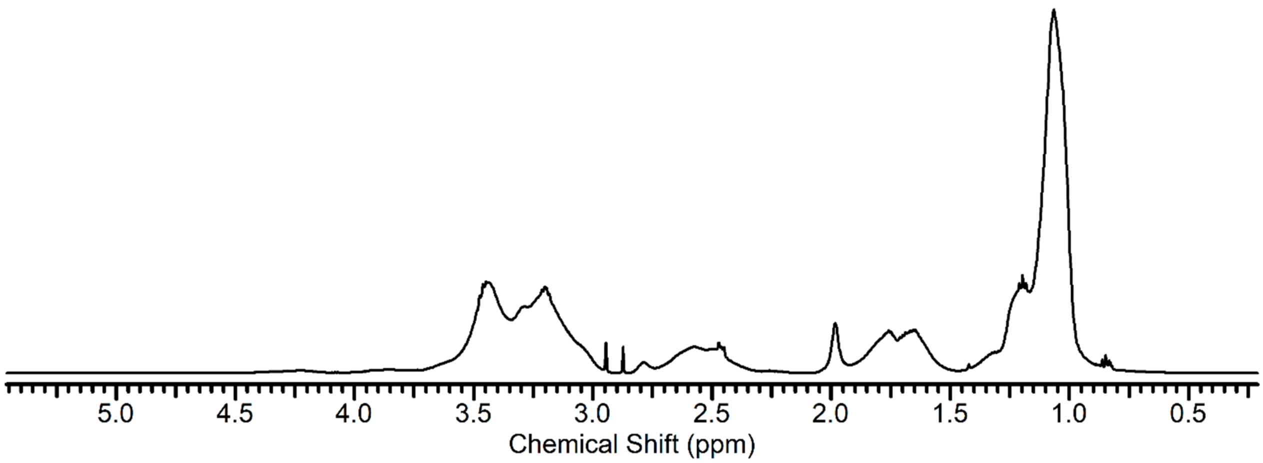


**Figure S3.** $^{1}$H NMR spectrum of *Sample **C*** P(DEAAm-*co*-GMA) copolymer (molar feed ratio AIBN:DEAAm:GMA = 1:190:10).

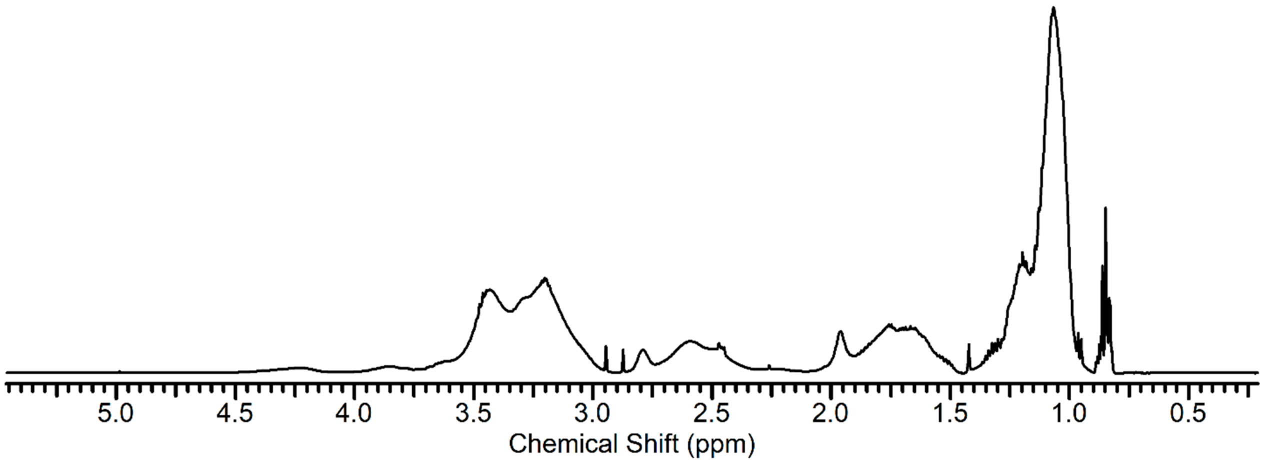


**Figure S4.** $^{1}$H NMR spectrum of *Sample **D*** P(DEAAm-*co*-GMA) copolymer (molar feed ratio AIBN:DEAAm:GMA = 1:180:20).

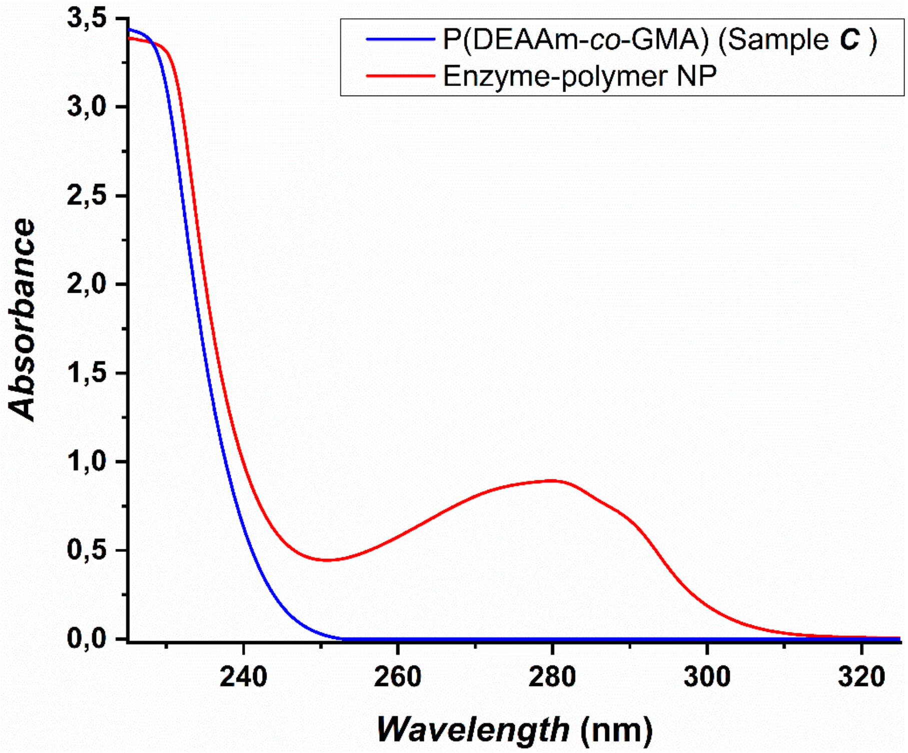


**Figure S5.** UV spectra of the P(DEAAm-*co*-GMA) (Sample *C*, *blue*) and the produced enzyme-polymer nanoparticle (*red*).

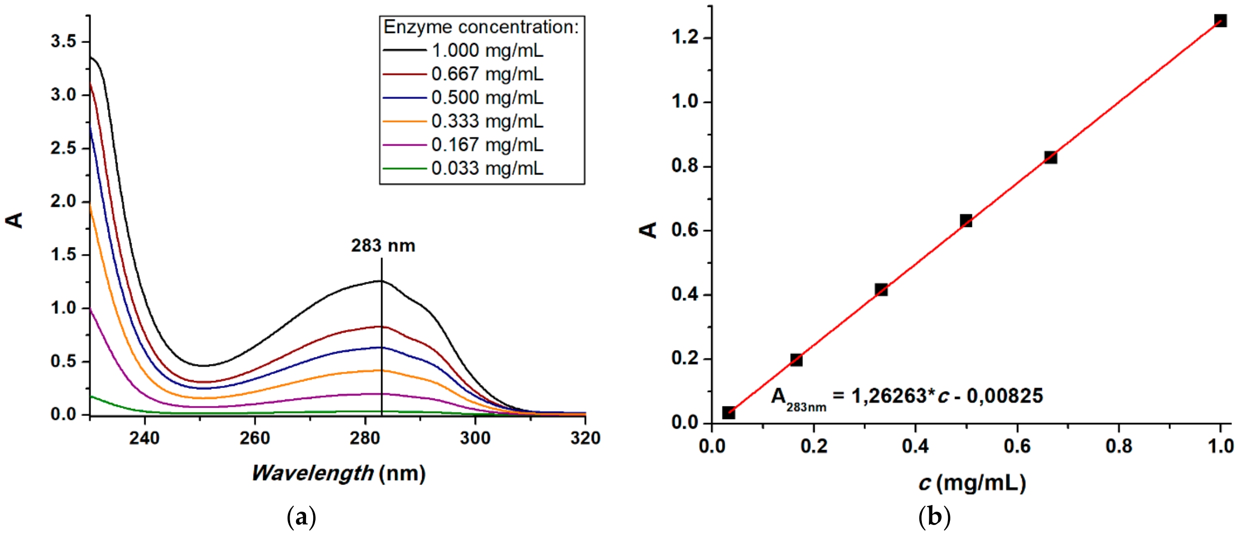


**Figure S6.** UV spectra of the α-chymotrypsin in the concentration range of 0.033-1 mg/mL (**a**) and the calibration curve fitted on the absorbance at 283 nm as a function of the enzyme concentration (**b**).

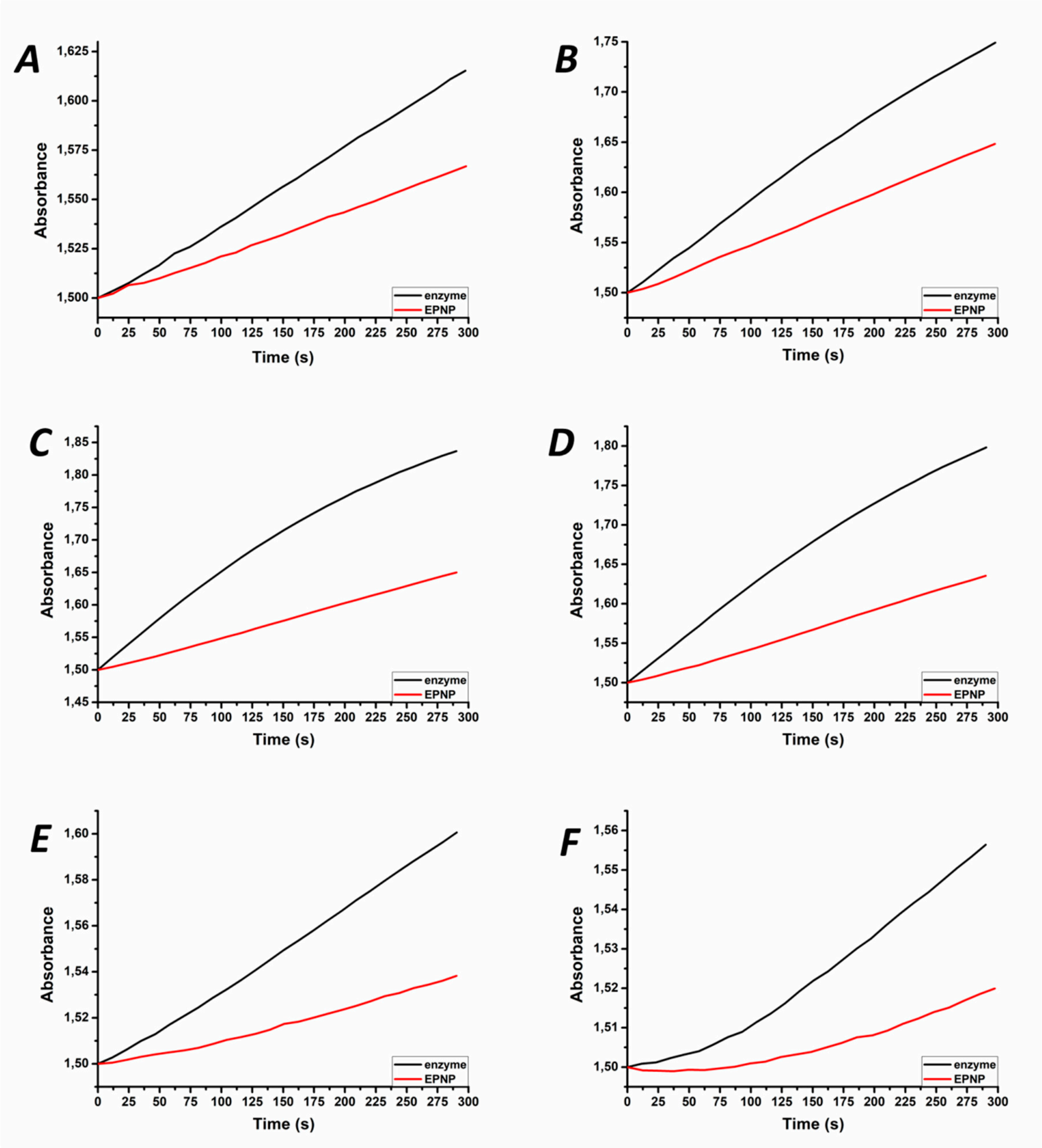


**Figure S7.** Representative enzymatic activity investigation curves of the absorbance measurement of the enzyme (black) and EPNP (red) in time in different pH solvents (pH = 6 (***A***); 7 (***B***); 7.4 (***C***); 7.8 (***D***); 8 (***E***); 9 (***F***)).

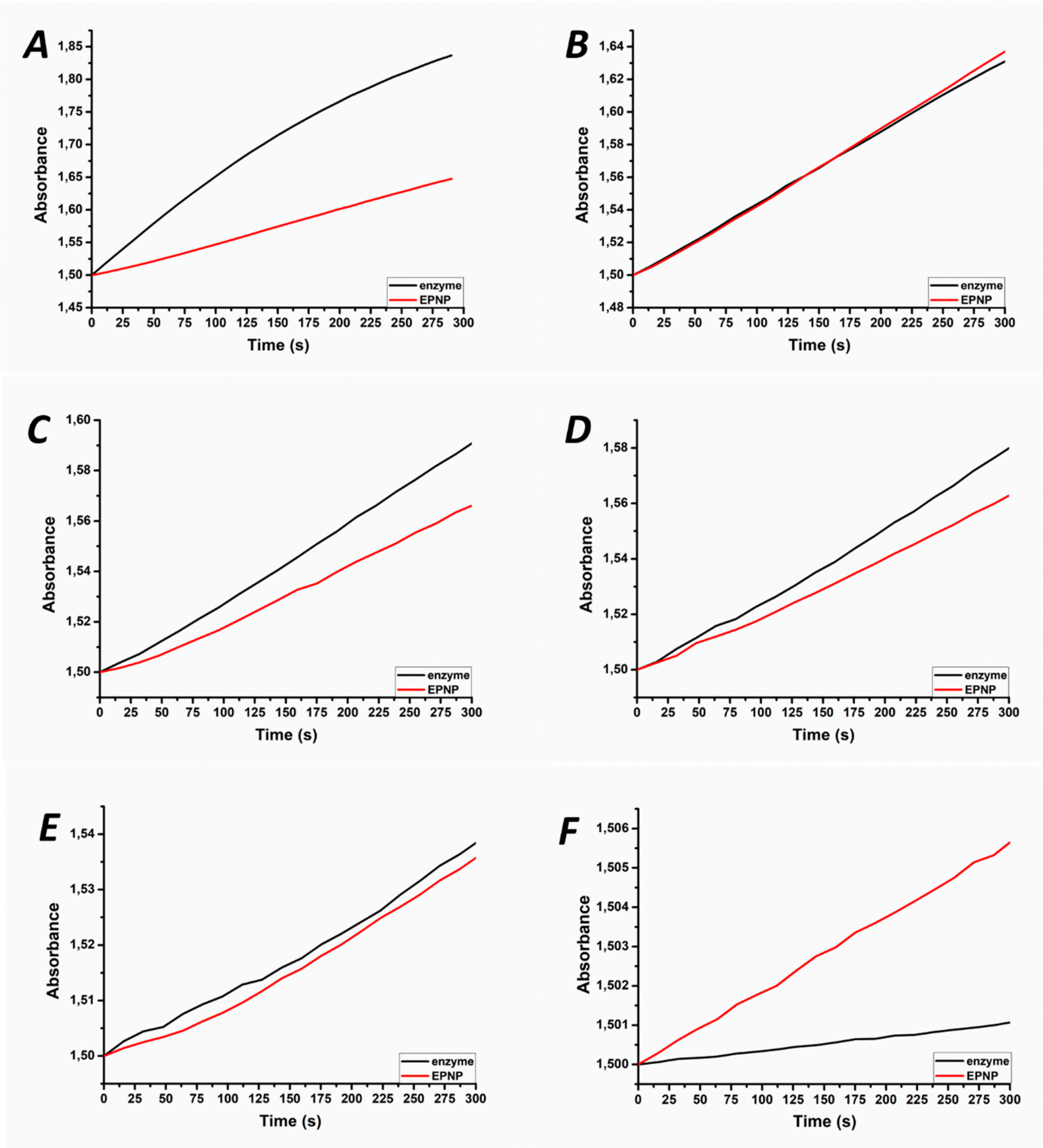


**Figure S8.** Representative curves of the activity measurements of the enzyme (black) and EPNP (red) in PBS buffer after thermostated at 45 °C for 0 min (***A***), 5 min (***B***), 15 min (***C***), 30 min (***D***), 60 min (***E***) and 120 min (***F***).
